# Cerebral Ultrasound at Term-Equivalent Age: Correlations with Neuro-Motor Outcomes at 12–24 Months Corrected Age

**DOI:** 10.3390/children12010030

**Published:** 2024-12-28

**Authors:** Adrian Ioan Toma, Vlad Dima, Lidia Rusu, Alexandra Floriana Nemeș, Bogdan Florin Gonț, Alexandra Arghirescu, Andreea Necula, Alina Fieraru, Roxana Stoiciu, Larisa Andrășoaie, Loredana Mitran, Claudia Mehedințu, Al Jashi Isam

**Affiliations:** 1 Life Memorial Hospital, 010719 Bucharest, Romania; adrian.toma@prof.utm.ro (A.I.T.); larisa.andrasoaie@s.utm.ro (L.A.); 2 Faculty of Medicine, University Titu Maiorescu, 040441 Bucharest, Romania; 3 Neonatology Department, Filantropia Clinical Hospital, 011132 Bucharest, Romania; 4 Regional Center of Public Health, 700465 Iasi, Romania; 5 Elias University Emergency Hospital, 011461 Bucharest, Romania; 6 Department of ENT, Carol Davila University of Medicine and Pharmacy, 050474 Bucharest, Romania

**Keywords:** premature neonates, TEA cerebral ultrasound, gross motor outcome, fine motor outcome, correlation, gyral folding, ventricular midbody, basal ganglia

## Abstract

**Background/Objectives:** Our research aimed to assess if correlations could be found between items evaluated at the cerebral ultrasound performed at term-equivalent age (TEA) and neuro-motor outcomes evaluated at 12 and 24 months of corrected age in a group of preterm infants. **Methods:** The following were assessed: the Levine Index, the diagonals of the lateral ventricles, the size of the ventricular midbody, the sinocortical distance, the width of the basal ganglia, the cortical depth at the level of the cingular sulcus and the maturation of the gyral folding. The neurologic evaluation was performed at 12 and 24 months of corrected age, according to the Amiel Tison neurologic examination, and the items from the calendar of motor acquisitions were used as outcome measures of the study—gross and fine motor subsets. The comparisons between the different groups were performed using the FANOVA test, with a statistically significant association for a *p* < 0.05. **Results:** The abnormal gross motor acquisitions at 12 months were significantly associated with an increased size of the ventricular midbody (*p* < 0.009) and a significantly decreased diameter of the basal ganglia (*p* < 0.011) on the TEA cerebral ultrasound. At 24 months, a significant association was found with increased size of the ventricular midbody (>10.33 mm) (*p* < 0.001), a decreased diameter of the basal ganglia (<12.9 mm) (*p* < 0.016), a decreased cortical depth (*p* < 0.021) and an immature gyral maturation pattern (*p* < 0.001). In the case of severely abnormal fine motor outcomes, at 12 months, there were statistically significant associations with an increased size of the ventricular midbody (*p* < 0.001) and an immature gyral folding pattern (*p* < 0.0180); at 24 months, significant associations were noted with the size of ventricular midbody (*p* < 0.001), a decreased diameter of the basal ganglia (*p* < 0.016), a decreased cortical depth (*p* < 0.021) and an immature gyration folding (*p* < 0.001). **Conclusions:** The abnormal gross and fine motor outcome in former premature infants at 12–24 months corrected age is significantly associated with abnormal findings in the head ultrasound examination performed at TEA reflecting both white matter (increased midbody distance) and grey matter (decreased diameter of the basal ganglia, decreased cortical depth and an immature gyration pattern) involvement.

## 1. Introduction

The encephalopathy of prematurity represents a complex disease, a frame used to explain the different but related grey and white matter lesions occurring in the brains of premature neonates [1,2]. The mechanisms are complex and, as mentioned above, involve both white and grey matter [1,2]. Probably, the initiating event is represented by the white matter injury, leading to the loss of the pre-oligodendrocytes [1]. According to theory and observational and experimental data, this leads to several dysmaturational events involving multiple structures [2]. Six scenarios were discussed [1,2]:

1. Pre-oligodendrocyte death resulting in decreased myelination and axonal dysmaturation leading to decreased cortical and thalamic development.
2. Axonal injury leading to the same consequences.
3. Thalamic injury leading to axonal dysmaturation, decreased myelination and decreased cortical and thalamic development.
4. Injury of the subplate neurons leading again to decreased myelination and cortical development.
5. Injury of the migrating GABA-ergic (GABA = gamma-amino-butyric acid) neurons that lead to decreased development of the upper cortical layers.
6. Primary injury that leads to secondary dysmaturation.

As can be observed, the structures involved are multiple not limited to the white matter and anatomical consequences are spread through the brain. As mentioned earlier, the central event is represented by the white matter injury with a variety of neuronal and axonal deficits involving the cerebral cortex and the thalamus [1].

The mechanisms of the lesions are complex and poorly understood. They involve a series of ischemic, infectious and inflammatory events occurring in a developing brain in the context of impaired regulation of the cerebral circulation resulting in a pressure passive circulation [3,4,5,6].

Even if, in the past, the belief has been that this is a pathology of the extremely preterm infants [7], and the small premature infants still represent the main population affected [8], there is more and more data to suggest these lesions occur also in premature infants over 30 weeks gestational age, though with certain particularities [9,10,11]. Even more, in the case of late preterm infants, the dysmaturation involves more grey matter structures, especially the cortex [11].

Most cases are asymptomatic in the neonatal period, and the diagnosis relies on imaging in all patients at risk [8]. For a long time, MRI has been considered the gold standard for the diagnosis of the effects of the encephalopathy of prematurity, especially in the white matter [12,13] and a superior value of the MRI compared to the head ultrasound was demonstrated [14]. More recent studies, using scoring systems, showed a good correlation between head ultrasound and MRI, both performed at term-equivalent age for neurologic deficits [15,16]. A good correlation between the ultrasound and MRI measurements of the same brain structures was demonstrated [17].

The scores assess both measured items and items that are visually evaluated by the observer [15,16]. The measured items evaluate, based on certain dimensions of the ventricles (the Levine Index, the diagonals of the anterior horn, the distance at the midbody of the ventricles) and the subarachnoid space, the consequences of the white matter loss [15,18], as markers of white matter volume loss at TEA [15,19]. Also, the involvement of the grey matter is evaluated by the measurements of the head of the caudate nucleus, the width of the basal ganglia and the cortical depth [15]. An important measure of the involvement of the grey matter is the degree of cortical maturation—gyration and cortical folding [15]. This item is also used by MRI studies [20] and is based on the already-known pattern of gyral maturation throughout gestation [21,22]. A delayed gyral maturation could be assessed also by ultrasound by using the same criteria as MRI [15] and was also correlated with long-term outcomes [15,16].

The long-term consequences of the encephalopathy of prematurity include motor deficits (from severe involvement in the form of spastic cerebral palsy to less severe forms of developmental coordination disorder) and various cognitive, behavioral, attention and socialization problems [8].

The most important neuro-motor consequence of the encephalopathy of prematurity is represented by cerebral palsy (CP), defined as a group of permanent disorders of the development of movement and posture [23]. According to a comprehensive population study, the prevalence of the disease is at 2–3% and decreasing in the general neonatal population, with low gestational age being an important risk factor [24]. Even if the overall prevalence is decreasing, the prevalence of the disease among premature infants remains constant (10–14%) [8]. White matter disorder remains the main risk factor for the occurrence of cerebral palsy [25]. The form of CP specific to former premature infants is a spastic bilateral CP with predominant involvement of the lower extremities [8], which is manifested predominantly by delayed gait or gait disturbances [26]. The occurrence of this complication could be explained by the topography of the white matter lesions—at the level of the motor pathways [8,16].

The second, more interesting and more difficult to explain neuro-motor pathology in former premature infants is represented by the developmental coordination disorder [8,26,27], defined as a chronic, usually permanent condition characterized by motor impairment that interferes with a child’s activities of daily living [27]. There are difficulties mentioned in gross and fine motor skills, not reaching the level of CP, balance, hand–eye coordination manual dexterity [8,27]. It is believed that 19/100 of preterm infants have a moderate form of DCD and 40/100 have a mild involvement [26].

Early identification of the former premature infants with a risk of motor deficit is essential to include them in early intervention programs [19]. Indeed, a recent meta-analysis, even if a definitive impact of early intervention on neurodevelopment could not be shown, could lead to an improvement of the cognitive and motor outcomes during infancy but not at preschool or school age [28]. Our group showed that early identification of the infants at risk by using the General Movements patterns can be effective, and by using an approach based on movement-induced therapy, an improvement was obtained at 12 weeks of corrected age [29].

Considering all of the above, our research aimed to assess if correlations could be found between the items evaluated at the cerebral ultrasound performed at TEA and neuro-motor outcomes evaluated at 12 and 24 months of corrected age in a group of preterm infants with gestational ages more than 30 weeks. Considering the involvement of both grey and white matter in the case of the encephalopathy of prematurity, we selected items that are known to be markers of white-matter volume loss (the Levine Index, diagonals of the ventricles and the distance at the midbody of the lateral ventricle and sinocortical width) and of grey matter loss and dysmaturation (cortical depth, gyral maturation and the diameter of the basal ganglia). The early identification of these patients by these simple means would allow a better prognostication for the families, a rapid inclusion in the early intervention programs and not at least a better understanding of the mechanisms involved in the occurrence of this complex disease.

## 2. Materials and Methods

Thirty-four premature infants with gestational ages between 30 and 34 weeks have been included in the study. They were part of the follow-up program at Life Memorial Hospital Bucharest.

The follow-up program for high-risk neonates at Life Memorial Hospital (today Medlife Medical Park) is one of the oldest in the country (established in 2010 and still functional). It is organized according to the national and international guidelines. The program’s structure consists of a network of specialists providing early diagnosis and intervention for the patients. The first visit is performed by the pediatric neurologist at 40 weeks of corrected age for premature neonates or at discharge from the hospital for term neonates. Two neurologic examinations are performed: the Amiel Tison neurologic examination for neonates [30]. Since 2023, Prechtl General Movements [31] examination has been added, but this is not the case in the patients in this study who were born in 2021–2022. During the first visit, a head ultrasound and hearing screening were performed. The next visits were conducted at 4 months, 7 months and 12 months. The Amiel Tison Neurologic examination was performed for 0–6 years [32]. In case of an abnormal exam, the patient was assigned to a physical therapy/kinetotherapy and re-habilitation specialist and re-assessed in 2–3 months to evaluate the efficacy of the therapy. From 12 months onwards—at 12-, 18- and 24-month visits—along with the neurologic evaluation, the patient was also referred to a psychologist for a Bayley III evaluation [33] and an MCHAT test for early diagnosis of autistic spectrum disorder at 18 months; in case of abnormalities noted at these tests, the results were discussed with the language therapy specialist and child psychiatrist and the therapy started accordingly. After 24 months, yearly visits were scheduled at 3, 4, 5 and 6 years of age.

The patients participating in the follow-up program are NICU graduates, according to the national and international guidelines:

- Premature neonates less than 35 weeks gestation.
- Neonates with perinatal asphyxia.
- Neonates with hyperbilirubinemia needing exchange transfusion.
- Term or preterm neonates requiring mechanical ventilation.
- Neonates with other central nervous system pathology (intracranial hemorrhages or infarctions—arterial or venous, CNS infections, seizures).
- Neonates with the neurologic screening examination (Amiel Tison neurologic optimality examination) with abnormal results.
- Neonates with sepsis.
- Neonates involved in studies with a follow-up component.

Usually, inborn patients are examined in the program, but consultations are opened for patients born in other centers.

The patients in the study were premature neonates who have been followed in the program until the age of 24 months (2 years) of corrected age, were born during 2022 and had 24 months of corrected age until November 1, 2024. To be part of the study, the patients should be premature infants born between 30 and 34 weeks of gestational age, have a complete neurologic examination and head ultrasound at 40 weeks of corrected age and complete the follow-up examination both at 12 and 24 months of corrected age. Forty patients met the criteria for a full neurologic exam and ultrasound at 40 weeks of corrected age, 36 were examined at 12 months of corrected age and 34 completed the examination (follow-up rate of 85%).

The procedures performed (cerebral ultrasound, neurologic examination) were part of the normal examination protocol for a premature infant at term-equivalent age (TEA) and 12 and 24 months of corrected age. The approval was obtained from the Ethics Committee of the hospital (Decision 13/5.11.2024). Also, all the parents of the patients involved in the study provided informed consent for participation.

### 2.1. Ultrasound Examination

The head ultrasound examination was performed at 40 weeks of corrected age/term-equivalent age (TEA).

A Vivid S60 (General Electrics—GE) was used to perform the examinations. There were two probes used—a micro-convex probe with a frequency of 5–7.5 MHz and a linear probe with a frequency of 7–12 MHz. To maintain the safety of the examination, the Mechanical Index (MI) and Thermal Index (TI) were maintained below 1.0 [34,35].

All the measurements were performed through the anterior fontanelle, in coronal, sagittal and para-sagittal sections.

As markers of white matter involvement, the following were measured:

- The Levine Index and the diagonals of the frontal horns of the lateral ventricles—measured in a coronal section at the level of the foramen of Moro [15,36,37]—a measure of frontal white matter loss [18];
- The size of the ventricular midbody—measured in a parasagittal plane [15,37]—a measure of peri-trigonal white matter loss [18];
- The sinocortical distance—a measure of the dilatation of the subarachnoid space [18]—measured in a coronal plane at the level of the foramen of Moro [15,36,37].

As markers of grey matter and cortical involvement, the following were measured:

- The width of the basal ganglia—measured on a coronal section at the level of the foramen of Moro [15,35,36]—as a marker of loss of central grey matter [37,38];
- The cortical depth of the cingular sulcus, measured in a para-sagittal plane, at the level of the segment of the cingular sulcus parallel to the colossal body [17,36,37];
- The gyral maturation pattern—evaluated in 3 sections—coronal at the level of the foramen of Moro—looking at the Sylvian fissure and the depth of the sulci: sagittal—looking at the frontal gyri and sulci and the presence of the central/marginal sulcus and parasagittal, temporal, at the level of the insula [39]. The cortical gyral maturation was assessed according to previously published scores [15,20], and to know patterns of sulcal and gyral maturation [21,22], divided into three categories:
  ○ Mature, normal pattern, corresponding to 38–40 weeks;
  ○ Moderately immature—corresponding to 34–36 weeks;
  ○ Immature—corresponding to less than 34 weeks.

The technique of the measurement and the sections used have been presented by our group in previous research in the case of the Levine Index, diagonals of the lateral ventricles, size of the ventricular midbody, sinocortical distance, the width of the basal ganglia and the cortical depth at the cingular sulcus [37].

**Figure 1:**
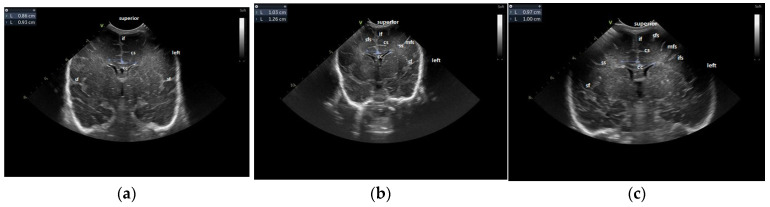
Maturation of the gyration–coronal section: (**a**) immature pattern; (**b**) moderately immature pattern; (**c**) normal patterns. Figure 1a: Immature pattern—only the main primary gyri are present—the smooth surface of the brain, no secondary gyri. Figure 1b: Moderately immature—the gyri are deeper than the previous figure—secondary gyri (ss) appear. Figure 1c: Normal pattern—secondary gyri present, increase in complexity of gyral folding. Legend: cc, corpus callosum; cs, cingulate sulcus; if, interhemispheric fissure; ifs, inferior frontal sulcus; mfs, middle frontal sulcus; sf, Sylvian fissure, sfs, superior frontal sulcus; ss, secondary sulcus.

**Figure 2:**
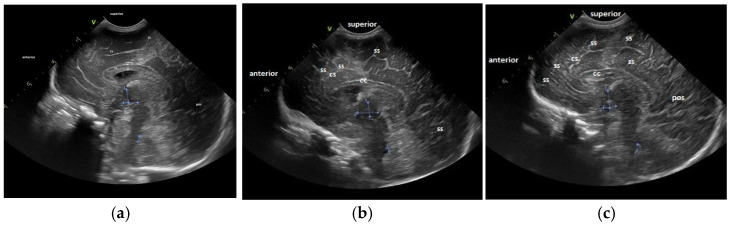
Maturation of the gyral pattern—sagittal section: (**a**) immature pattern; (**b**) moderately immature pattern; (**c**) normal pattern. (**a**) Immature pattern—just primary gyri present. Smooth surface frontal and parietal lobes. (**b**) Moderately immature pattern—secondary sulci (ss) become visible in the frontal and occipital lobes. (**c**) Normal pattern—secondary sulci become visible in frontal, parietal and occipital lobes. Increase in complexity of the gyral pattern compared to b. Legend: cc, corpus callosum; cs, cingulate sulcus; pos, parieto-occipital sulcus; ss, secondary sulcus (sulci).

**Figure 3:**
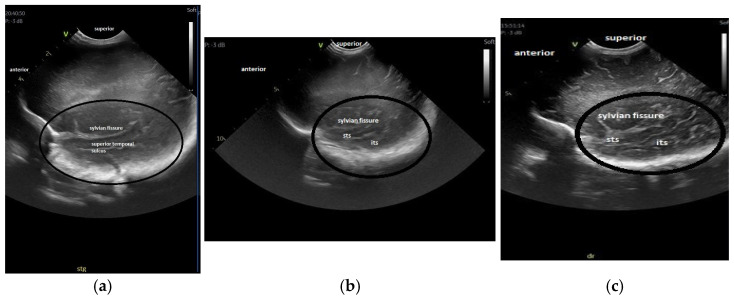
Maturation of the gyration—parasagittal opercular section: (**a**) immature pattern; (**b**) moderately immature pattern; (**c**) mature pattern The region of interest, part of the temporal lobe is encircled in all the figures. Figure 3a: Immature pattern—only the superior temporal sulcus is visible, there are no inferior temporal sulcus or secondary or tertiary sulci and the surface is smooth. Figure 3b: Moderately immature—both superior and inferior temporal sulci are visible and there is a beginning of ramification in secondary sulci. Figure 3c: Mature pattern—the primary temporal sulci are visible and secondary sulci and ramifications can be seen. Legend: its, inferior temporal sulcus; sts, superior temporal sulcus.

**Figure 4:**
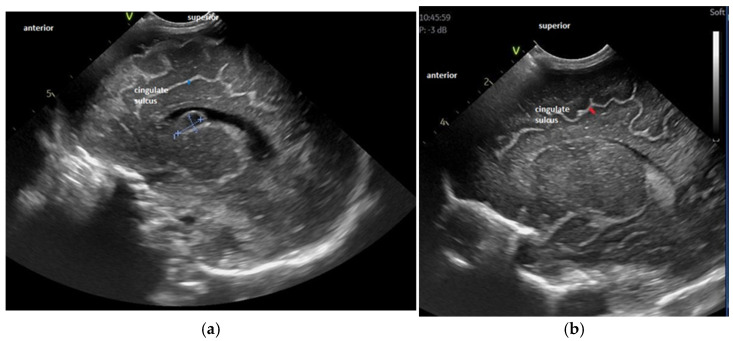
Examples of decreased (**a**) and normal (**b**) cortical depth at the level of the cingular sulcus—parasagittal section—see orientation mark in the figure. Figure 4a: Cortical depth 1.6 mm—30 weeks GA premature infant examined at term-equivalent age, with white matter disorder and a post-GMH (germinal matrix hemorrhage) cyst at the level of the caudothalamic groove—depth 1.6 mm. Figure 4b: Normal sulcal depth in 33 weeks GA premature infant examined at term-equivalent age, depth 2.3 mm.

For the maturation of gyral patterns, examples of mature, moderately immature and immature patterns are shown in Figure 1, Figure 2 and Figure 3. Figure 4 displays an example of cortical depth at the level of the cingular sulcus for normal and abnormal measurements.

### 2.2. Neurologic Evaluation

The patients have been examined as a part of the follow-up program for high-risk NICU graduates, at Life Memorial Hospital Bucharest, at different time intervals, according to the local and international guidelines [40,41,42,43].

The Amel Tison neurologic examination for newborns [30] has been used for the evaluation of patients at term-equivalent age (TEA). At 12 and 24 months of corrected age, Amiel Tison neurologic examination for children 0–6 years has been used [32]. The items from the calendar of motor acquisitions—both gross motor and fine motor—have been used as outcome measures of the study and were graded according to the examination chart Ș normal for the age; moderately abnormal and two severely abnormal (see Table 1—12 months corrected age; Table 2—24 months corrected age)—developed from reference [32].

### 2.3. Statistical Analysis

The data were analyzed by an independent statistician, to eliminate any interpretation bias. The ANOVA test was used to investigate the statistical relations between different parts of a patient group or the different characteristics and interdependence links between the variables:

The following indicators (described by the ANOVA test) were used:

- Indicators for mean values: mean, median, module and the maximum and minimum values.
- Indicators for dispersion: the standard deviation and the deviation coefficient.
- The Skewness test (−2 < *p* < 2) that validates the normal distribution of the series of values; it is used when the variable is a continuous one [44].

To investigate the presence of a statistically significant difference between two or more groups, according to the distribution of the series of values, for a significance level >95%, the following tests were applied:

- The *t*-Student test—a parametric test that compares the mean values for two groups with a normal distribution.
- The F (ANOVA) test—which is used in the case of comparison of 3 or more groups with a normal distribution, adding the Turkey post hoc correction to offer the largest difference between two means.
- The χ2 test—a non-parametric, qualitative test that compares the distribution of frequencies.
- The Kruskal–Wallis correlation that compares ordinal variables—3 or more groups.
- The Pearson(r) correlation coefficient—correlation between 2 variables within the same sample—the direct/indirect correlation is indicated by the sign of the coefficient (+/−) [45].

## 3. Results

### 3.1. Characteristics of the Population

The repartition of gender and gestational ages of the patients are provided in Table 3. There may be a slight predominance of females, especially at lower gestational ages.

### 3.2. Gross Motor Outcome and Relation to Cerebral Ultrasound Measurements at TEA

At the examination at 12 months of corrected age, 19/34 infants presented with normal development, 5/34 were moderately abnormal (independent sitting but not walking yet) and 10/34 could not sit or walk independently (severely abnormal examination). The abnormal gross motor acquisitions were significantly associated with an increased size of the ventricular midbody (*p* < 0.009) and a significantly decreased width of the basal ganglia (*p* < 0.011) (Table 4).

At the 24-month corrected age examination, 30/34 patients presented with a normal exam and 4/34 with a severely abnormal exam (not walking at all—alone or with assistance). The severely abnormal gross motor acquisitions at 24 months of corrected age were significantly associated with an increased size of the ventricular midbody (>10.33 mm) (*p* < 0.001) and a decreased width of the basal ganglia (<12.9 mm) (*p* < 0.016) and also with a decreased cortical depth (*p* < 0.021) and an immature gyral folding pattern (*p* < 0.001) (Table 5).

### 3.3. Fine Motor Outcome and Relation with Cerebral Ultrasound Measurements at TEA

In the case of the fine motor outcome, the acquisitions were within normal limits at 12 months of corrected age in 20/34 patients, moderately abnormal in 7/34 and severely abnormal in 7/34 patients. At this age, abnormal fine motor acquisitions were significantly associated with an increased size of the ventricular midbody (*p* < 0.001) (Table 6 and an immature gyration folding (*p* < 0018) (Table 7)—on the head ultrasound exam at TEA.

At the examination at 24 months of corrected age, 30 patients had the exam within normal limits and 4/34 had a severely abnormal exam. The severely abnormal exam was statistically significantly associated with an increased size of the ventricular midbody (*p* < 0.001), a decreased diameter of the basal ganglia (*p* < 0.016), a decreased cortical depth (*p* < 0.021) (Table 6) and an immature gyration folding (*p* < 0.001) (Table 7).

## 4. Discussion

Our research demonstrated that correlations were present between head ultrasound findings at TEA and the neuro-motor outcomes at 12–24 months. The measurements that correlated with both gross and fine motor outcomes were considered to be markers of withe matter [15,18,35] or grey matter [15,20] injuries.

In the case of the gross motor outcome, the main ultrasound correlation of the severely affected infants (not able to walk independently) has been an increased size of the ventricular midbody. This finding could be explained by the fact that the enlargement of the ventricular midbody is considered to be a marker of periventricular white matter injury [15,18], and the motor deficit resulting from the white matter injury in the preterm neonates results in a pattern of cerebral palsy characterized by gait disturbances related to the lesions of specific corticospinal pathways leading to the legs due to the topographic distribution of the fibers [7]. No correlation has been found between abnormal motor outcome and the Levine Index or the diagonals of the lateral horns—both markers of frontal white matter loss [18]. Based on these findings, we could speculate that in our group and perhaps in larger (≥30 weeks) infants, the peri-trigonal white matter is more affected than the frontal white matter. The interesting findings concerning gross motor outcomes are the presence in the severely affected neonates of a decreased diameter of the basal ganglia at 12 and 24 months and an association with a decreased cortical depth and an immature gyral folding at the TEA head ultrasound examination in the children with an absent gait at 24 months of corrected age. The items listed above are grey matter structures—both deep (basal ganglia) and cortical–sulcal width and gyral folding. Those findings could give us an insight into the pathophysiology of the motor deficit in former premature infants in the broader context of the encephalopathy of prematurity. Indeed, the disease that probably starts with the lesions in the pre-oligodendrocytes involves, by dysmaturation, the neurons in the basal ganglia and cortex [1,2,7] as explained also in the introduction. So, it should not be surprising that these structures are affected also in the most severe cases of white matter injury that leads to a motor deficit. In addition, the more the cortical structures are affected, the greater the deficit—an immature gyral pattern and a decreased cortical depth are observed in the former premature infant with a severe gross motor deficit at 24 months of corrected age. This is supplementary proof that it is not only the white matter that is affected in the case of infants with CP. Indeed, it is not only the interruption of the motor pathways involved in the motor deficit in patients affected in the neonatal period but also the absence of fine-tuning and modulation of the movements given by the cortex and the basal ganglia [7,45].

The association is more obvious in the case of fine motor outcomes. A severely abnormal motor outcome at 12 months (absence of pincer grasp) was associated with markers of white matter injury and injury to the motor pathways (midbody distance) and also markers of cortical dysmaturation (immature gyral folding) [7,14,15]. Again, the relation of a severely abnormal outcome with markers of cortical involvement is stronger at 24 months of corrected age: There is a strong association with decreased width of the basal ganglia and cortical immature gyral pattern and decreased cortical depth noted at the TEA head ultrasound. This represents probably a result of the stronger relation between fine motor components (grasp and manipulation of the objects with hands) and the fine cortical tuning of the movements [45].

The finding of a better correlation of markers of grey matter involvement than those of white matter involvement with the neuromotor outcome in our group confirms the previous findings that dysmaturation involves in late-preterm infants more grey matter than white matter structures (see also Introduction) [11].

We believe that the main strengths of this study lie in its design, which is simple and avoids selection and interpretation biases, and in the inclusion of variables assessed for predicting the motor outcomes both those related to white and grey matter involvement. The ultrasound data are gathered at the TEA exam, and the measurements are performed after this, blinded, to avoid interpretation bias [46]. Also, avoidance of the bias has been accomplished by the statistical analysis performed by an independent statistician, from another center, who received the database and the research questions. Also, a strong point could be regarded as the inclusion of the maturation of gyral folding as a finding associated with gross and fine motor outcomes; this is one of the few studies we are aware of evaluating the link between these variables [14,15].

The variables noted at the head ultrasound exam are evaluated in standard sections and are very simple to measure [15,34,39]; in addition, there are standardized measurements for all of these [35]. These items are part of different scoring systems [15,16] and have been used by our group previously for assessing the correlation of head ultrasound findings with General Movements items [36]. There is, indeed, one item that is evaluated by the physician performing the ultrasound and its evaluation could be subjective—that is the pattern of gyral maturation. Measures have been taken to avoid a subjective evaluation of this item as much as possible. First, the evaluation has been performed blindly, on standardized sections, after the acquisition of the images to avoid interpretation bias. Second, the scoring system for the gyral maturation was established according to the scores already in use [15,20] considering the maturation of the gyral folding related to the gestational age [21,22]. Also, we believe that a strong point of our study is including larger premature neonates (gestational ages > 30 weeks) in the research, unlike previous studies. Even if, as discussed in the introduction, the main neuro-motor risks are seen in premature infants less than 28 weeks gestational age, the moderate and late preterm infants represent a group with important risks [8,9,10] and are less investigated compared to other groups. So, even if this could be seen as a weak point, we consider that evaluating this category of neonates adds value to our findings.

The weak points of the study, in our view, could be represented by the small number of patients, the monocentric design and the absence of the MRI examinations at TEA to be compared with the ultrasound examinations. The number of patients is indeed a small one, and the observations are made only in one follow-up program. This is why we advise caution when generalizing the results of this research in the whole premature neonate population, and we wish that the results be validated in larger multi-centric studies. Regarding the MRI examinations, at this moment, there is controversy about having a head MRI as a standard for premature infants at TEA, with publications both for and against it [47,48]. In addition, as shown in the introduction (see above), previous studies have demonstrated the concordance between the predictive values of MRI and head ultrasound regarding the prognosis in former premature infants if the examinations were performed at TEA [15] and comparable values for the measurements performed on head ultrasound and MRI [17].

Besides underlining the prognostic value of TEA head ultrasound in former premature neonates in anticipating gross and fine motor deficits and adding information about the complex pathophysiology of the encephalopathy of prematurity, this study raised several questions as further directions of study. The first question is if correlations can be found also between language and cognitive development and TEA head ultrasound findings. The second question is if later motor outcomes could be predicted also with the same parameters, and the third is if a simple score could be developed starting from this correlation as an outcome of a larger multicentric study involving more premature infants. These could be considered further directions of study for our and other research groups.

## 5. Conclusions

The abnormal gross and fine motor outcomes in former premature infants at 12–24 months of corrected age are significantly associated with abnormal findings in the head ultrasound examination performed at TEA reflecting both white matter (increased midbody distance) and grey matter (decreased diameter of the basal ganglia, decreased cortical depth and an immature gyration pattern) involvement. These findings could allow an early and better prediction of the outcomes, timely identification of the infants at risk and inclusion of the patients in early intervention programs and could also provide an insight into the complex pathogenesis of the neuro-motor dysfunction in the case of encephalopathy of prematurity. The result also shows us the importance of looking not only at the central structures (ventricles basal ganglia and thalami) in the premature neonates at TEA but also at the cortex and patterns of gyral folding; finding anomalies or immature patterns in these structures could have also prognostic implications.

Caution should be used when generalizing these findings to the general population +of premature neonates due to the small sample size and the monocentric nature of the study. The results of the research could, though, serve as a base for further larger multicentric studies performed to validate our hypotheses and results.

## Figures and Tables

**Table 1:** Outcome measures of the study—gross and fine motor acquisitions—12 months of corrected age developed based on reference [32].

	Normal	Moderately Abnormal	Severely Abnormal
Gross motor			
Independent sitting	Yes	Yes	No
Independent walking	Yes	No	No
Fine motor			
Finger grasp	Finger grasp	Palmar grasp	No grasp at all—asymmetry
Letting a cube in the cup	Yes	No	No
Tower of 3 cubes	Not evaluated	Not evaluated	Not evaluated

**Table 2:** Outcome measures of the study—gross and fine motor acquisitions—24 months of corrected age developed based on reference [32].

	Normal	Moderately Abnormal	Severely Abnormal
Gross motor			
Independent sitting	Yes	Yes	No
Independent walking	Yes	Walks with help	No
Fine motor			
Finger grasp	Finger grasp	Finger grasp	No grasp at al/palmar grasp
Letting a cube in the cup	Yes	No	No
Tower of 3 cubes	Yes	No	No

To be allocated to the normal group, the patient must have fulfilled all the criteria. Also, to be allocated to the severely affected group, the patient must fulfill all the criteria—for example, for the gross motor group—at 12 months—the patient would not sit independently and not walk, and at 24 months of corrected age, the patient should not be able to walk independently.

**Table 3:** Gestational age and gender repartition of the patients.

Weeks GA	Male	Female	Total
30	0	3	3
31	1	0	1
32	6	8	14
33	2	4	6
34	5	5	10
Total	14	20	34

**Table 4:** Levine Index, LV short axis, LV long axis, midbody LV, basal ganglia width, sinocortical width and cortical depth in relation to gross motor outcome at 12 and 24 month corrected age.

Parameters	Gross Motor 12 Months			*p*-Value for F_ANOVA_ Test
	Normal (n = 19)	Moderately Abnormal (n = 5)	Severely Abnormal (n = 10)	
Levine Index	10.84 ± 2.20	12.48 ± 0.65	11.61 ± 2.81	0.326
LV short axis	4.55 ± 1.39	5.70 ± 1.83	5.70 ± 1.83	0.220
LV long axis	12.92 ± 3.31	15.46 ± 2.44	14.47 ± 3.85	0.251
Midbody LV	3.15 ± 1.06	5.50 ± 2.17	5.73 ± 3.50	0.009
Basal ganglia width	16.54 ± 1.44	17.56 ± 1.18	14.92 ± 2.09	0.011
Sinocortical width	2.77 ± 1.18	3.90 ± 1.36	3.01 ± 0.96	0.162
Cortical depth	1.61 ± 0.44	1.80 ± 1.17	1.31 ± 0.63	0.320
**Parameters**	**Gross Motor 24 Months**			***p*-Value for F_ANOVA_ Test**
	**Normal (n = 30)**		**Severely Abnormal (n = 4)**	
Levine Index	10.42 ± 2.37		12.20 ± 1.30	0.253
VL short axis	4.90 ± 1.68		5.77 ± 1.20	0.435
VL long axis	12.87 ± 3.27		14.97 ± 4.26	0.388
Midbody VL	3.29 ± 1.35		**10.33 ± 1.53**	**0.001**
Basal ganglia width	16.16 ± 1.65		**12.90 ± 1.82**	**0.016**
Sinocortical width	3.09 ± 1.09		3.67 ± 1.53	0.483
**Cortical depth**	**2.09 ± 0.87**		**0.63 ± 0.40**	**0.021**

Legend: LV, lateral ventricle.

**Table 5:** Gyral maturation in relation to gross motor outcome at 12 and 24 months of corrected age.

Gross Motor	Gyration			*p*-Value for F_ANOVA_ Test
Gross Motor 12 months	Normal (n = 15)	Moderately Immature (n = 15)	Immature (n = 4)	
Normal	9 (60.0%)	10 (66.7%)	-	0.070
Moderately abnormal	3 (20.0%)	2 (13.3%)		
Severely abnormal	3 (20.0%)	3 (26.7%)	4 (100%)	
MG 24 months	a (n = 15)	b (n = 15)	c (n = 4)	
Normal	15 (100%)	15 (100%)	-	0.001
Severely abnormal	-	-	4 (100%)	

Legend: LV, lateral ventricle.

**Table 6:** Levine Index, LV short axis, LV long axis, midbody LV, basal ganglia width, sinocortical width and cortical depth in relation to fine motor outcomes at 7, 12, 18, 24 months.

Parameters	Fine Motor 12 Months			*p*-Value for F_ANOVA_ Test
	Normal (n = 20)	Moderately Abnormal (n = 7)	Abnormal (n = 7)	
Levine Index	10.95 ± 2.44	10.80 ± 2.01	12.84 ± 1.47	0.133
LV short axis	4.83 ± 1.75	4.47 ± 1.75	6.00 ± 1.46	0.182
LV long axis	13.37 ± 3.66	12.97 ± 2.67	15.60 ± 3.14	0.273
Midbody LV	3.45 ± 1.20	3.56 ± 2.35	7.27 ± 3.19	0.001
Basal ganglia width	16.62 ± 1.44	16.36 ± 2.03	14.90 ± 2.24	0.095
Sinocortical width	2.61 ± 0.95	3.64 ± 1.41	3.51 ± 1.20	0.054
Cortical depth	1.75 ± 0.63	1.43 ± 0.51	1.00 ± 0.59	0.054
**Parameters**	**Fine Motor 24 months**			***p*-Value for F_ANOVA_ Test**
	**Normal (n = 30)**		**Abnormal (n = 4)**	
Levine Index	10.42 ± 2.37		12.20 ± 1.30	0.253
VL short axis	4.90 ± 1.68		5.77 ± 1.20	0.435
VL long axis	12.87 ± 3.27		14.97 ± 4.26	0.388
Midbody VL	3.29 ± 1.35		10.33 ± 1.53	0.001
Basal ganglia width	16.16 ± 1.65		12.90 ± 1.82	0.016
Sinocortical width	3.09 ± 1.09		3.67 ± 1.53	0.483
Cortical depth	2.09 ± 0.87		0.63 ± 0.40	0.021

Legend: LV, lateral ventricle.

**Table 7:** Gyration about the fine motor outcome at 12, 18, 24 months.

Fine Motor	Gyration			*p*-Value for F_ANOVA_ Test
Fine Motor 12 Months	Normal (n = 15)	Moderately Immature (n = 15)	Immature (n = 4)	
Normal	8 (53.3%)	12 (80.0%)	-	0.018
Moderately abnormal	7 (26.7%)	0		
Abnormal	0	3 (13.3)	4 (75.0%)	
Fine motor 24 months	Normal (n = 15)	Moderately immature (n = 15)	Immature (n = 4)	
Normal Abnormal	15 (100%) -	15 (100%) -	- 4 (100%)	0.001

Legend: LV, lateral ventricle.

## Data Availability

The anonymized database is available as an Excel file upon request at adrian.toma@prof.utm.ro.
